# Moderate Salinity Stress Increases the Seedling Biomass in Oilseed Rape (*Brassica napus* L.)

**DOI:** 10.3390/plants12081650

**Published:** 2023-04-14

**Authors:** Beini Chen, Xiaobo Bian, Mengxin Tu, Tao Yu, Lixi Jiang, Yunhai Lu, Xiaoyang Chen

**Affiliations:** 1 Institute of Crop Science, Jinhua Academy of Agricultural Sciences, Zhihe Road 1158, Jinhua 321017, Chinaytzjjh@163.com (T.Y.); 2 Institute of Crop Science, Zhejiang University, Yu-Hang-Tang Road 866, Hangzhou 310058, China

**Keywords:** *Brassica napus*, salinity stress, seedling biomass, RNA-seq, shoot apical meristem

## Abstract

Oilseed rape (*Brassica napus* L.), an important oil crop of the world, suffers various abiotic stresses including salinity stress during the growth stage. While most of the previous studies paid attention to the adverse effects of high salinity stress on plant growth and development, as well as their underlying physiological and molecular mechanisms, less attention was paid to the effects of moderate or low salinity stress. In this study, we first tested the effects of different concentrations of NaCl solution on the seedling growth performance of two oilseed rape varieties (CH336, a semi-winter type, and Bruttor, a spring type) in pot cultures. We found that moderate salt concentrations (25 and 50 mmol L^−1^ NaCl) can stimulate seedling growth by a significant increase (10~20%, compared to controls) in both above- and underground biomasses, as estimated at the early flowering stage. We then performed RNA-seq analyses of shoot apical meristems (SAMs) from six-leaf-aged seedlings under control (CK), low (LS, 25 mmol L^−1^), and high (HS, 180 mmol L^−1^) salinity treatments in the two varieties. The GO and KEGG enrichment analyses of differentially expressed genes (DEGs) demonstrated that such a stimulating effect on seedling growth by low salinity stress may be caused by a more efficient capacity for photosynthesis as compensation, accompanied by a reduced energy loss for the biosynthesis of secondary metabolites and redirecting of energy to biomass formation. Our study provides a new perspective on the cultivation of oilseed rape in saline regions and new insights into the molecular mechanisms of salt tolerance in *Brassica* crops. The candidate genes identified in this study can serve as targets for molecular breeding selection and genetic engineering toward enhancing salt tolerance in *B. napus*.

## 1. Introduction 

Soil salinity is a serious and global threat to agricultural production and sustainable development [1,2]. A recent report revealed that over one billion hectares of land are currently affected by soil salinity, representing about 7% of the earth’s land surface [3]. Moreover, this problem is persistently spreading and growing over two million hectares per year [4]. High salt concentration in soil is harmful to plants due to the alteration of osmotic potential gradients and the consequent inhibition of some cellular functions, such as photosynthesis [5,6]. Therefore, salt stress adversely influences plant growth and development during the entire growth period of the plant through inducing physiological drought, ion toxicity, oxidative stress, nutrient imbalance, etc., and causes considerable yield losses [5,6,7]. Understanding the molecular and physiological mechanisms of plant response and adaptation to salt stress is crucial for genetic improvement and precise breeding of crops with a higher ability to tolerate salinity stress.

In the long course of evolution, plants have evolved to build complex and diverse mechanisms coping with salinity stress, including physiological responses, oxidative stress (ROS scavenging), salt stress sensing and signaling, organellar stress, ion homeostasis, hormonal and gene expression regulation, metabolic changes, etc. [8,9,10,11]. Considerable progress has been made in salinity stress research, and numerous genes or pathways in response to salinity stress have been identified through multi-omics methods in various plants, such as *Arabidopsis* [12,13], rice [14], sorghum [15], wheat [16], barley [17], cotton [18], sugar beet [19], and guar [20]. These salt stress-involved genes can be categorized into several functional groups, such as genes for photosynthetic enzymes, synthesis of compatible solutes, vacuolar-sequestering enzymes, and radical-scavenging enzymes. 

Oilseed rape (*Brassica napus* L.) is an important and widely cultivated crop throughout the world. It is ranked second (after soybean) among oil seed crops cultivated for edible oil for humans, and seed cake for animal consumption [21]. It is an allopolyploid plant species (AACC, 2n = 38), considered to have originated relatively recently (~6700–12,000 years ago) from a small number of natural hybridization events in Europe between two diploid progenitor species, *B. rapa* L. (AA, 2n = 20) and *B. oleracea* L. (CC, 2n = 18) [22,23,24]. It has adapted to diverse climate zones and latitudes by forming three main ecotype groups, namely winter, semi-winter, and spring types [25]. Oilseed rape came into cultivation ~400 years ago in Europe and initially formed a winter type and a spring type growing in distinct environments, and then was introduced into China in the 1930s–1940s, followed by crossing with the local *B. rapa* to give rise to the semi-winter type [26]. So, the oilseed rape cultivated in China is primarily semi-winter, requiring a shorter vernalization period to flower than the winter type. Today, oilseed rape is the largest oilseed crop in China and accounts for about 20% of world production [27].

During its growth, oilseed rape suffers from various abiotic stresses, including salinity stress [28]. Oilseed rape can tolerate moderate salt stress [29]. However, high salinity stress can seriously affect germination, root development, nutrient uptake, and photosynthesis during the early growth stage and plant growth, ultimately reducing the productivity of oilseed rape [28,29,30,31,32,33]. A diversity of salt tolerance capacity was observed among oilseed rape varieties, and this allowed the identification of genetic loci (QTLs), candidate genes, or genes in the genome of *B. napus* conferring the variations of salt tolerance [34,35,36,37,38,39]. While most of this previous research paid special attention to the effects of high salinity stress on plant growth and development, as well as their underlying physiological and molecular mechanisms, less attention was paid to the effects of moderate or low salinity stress. 

In the present study, we used two oilseed rape varieties (a spring type and a semi-winter type) as the experimental material and analyzed the effects of differently concentrated salt solutions on their seedling growth parameters. We found that moderate salinity stress can increase the seedling biomass in both of the two tested varieties. We then performed RNA-seq analyses with the objective to identify candidate genes that may be involved in stimulating seedling growth under moderate salinity stress conditions. This study provides a different perspective on the cultivation of oilseed rape and new insights into the molecular mechanisms of salt tolerance in *Brassica* crops.

## 2. Results

### 2.1. Response of Oilseed Rape Seedlings to the Treatments of Different Salinity Levels

**Figure 1:**
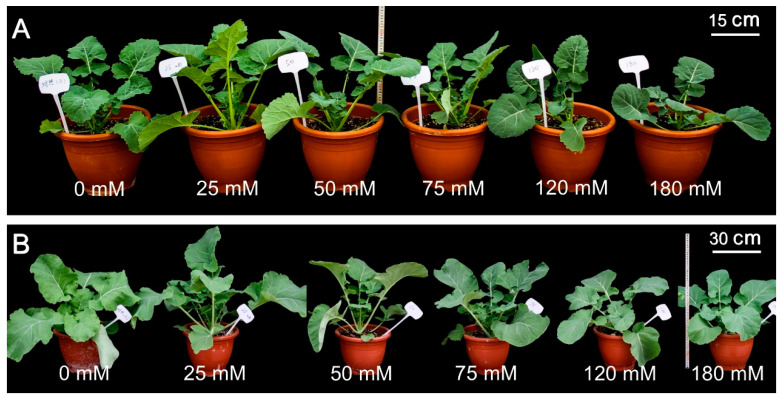
Effects of different salinity levels on the growth performance of two *Brassica napus* varieties, CH336 (semi-winter type) and Brutor (spring type), at six-leaf stage in pot cultures. (**A**). Semi-winter type variety CH336, Bar = 15 cm, (**B**). spring type variety Brutor, Bar = 30 cm.

Figure 1 shows the six-leaf-aged seedlings of two *B. napus* cultivars, CH336 (semi-winter type) and Brutor (spring type), in pot cultures under treatments of different salinity levels (i.e., 0, 25, 50, 75, 120, and 180 mmol L^−1^ NaCl). The data for the plant height at the six-leaf stage, and those for the aboveground fresh weight, underground fresh weight, aboveground dry weight, underground dry weight, and specific leaf weight (SLW) at the early flowering stage were recorded and summarized in Table 1. In general, the spring-type variety, Brutor, showed larger seedling sizes than the semi-winter-type variety, CH336 (2 to 3 times more as revealed by biomass values), and the growth performances of the two varieties were both significantly affected, depending on the tested salinity level (Table 1). Along with the gradual increase in NaCl concentration from 0 to 180 mmol L^−1^, the biomass of both above- and underground parts increased first, reached a peak at 25~50 mmol L^−1^, and then decreased in both varieties (Table 1). The lower levels (25 and 50 mmol L^−1^) of salinity treatment can significantly increase the seedling biomass by 10~20% (aboveground biomass), while the higher salinity levels (120 and 180 mmol L^−1^) can significantly decrease the seedling biomass by 20~40% compared with the control values (Figure 1, Table 1).

Specific leaf weight (SLW, leaf mass per area), as a leaf trait, was calculated for each of the six salinity levels in both varieties (Table 1). Under the control conditions, the specific leaf weight of the semi-winter-type variety, CH336, was significantly higher than that of the spring-type variety, Brutor (4.89 vs 3.86). Along with the increase in the salinity levels from 50 to 180 mmol L^−1^, the specific leaf weight showed an insignificant increase of 5~10% compared to the controls in CH336. However, the specific leaf weight increased by ~20% (compared to the controls) at the salinity levels of 75 mmol L^−1^ and 120 mmol L^−1^ in Brutor.

**Figure 2:**
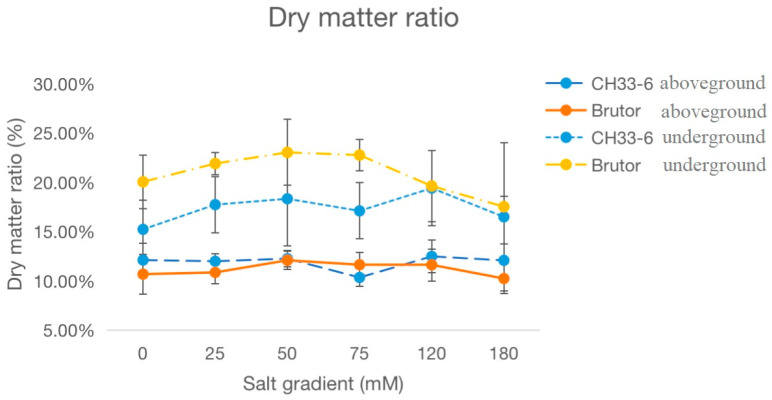
Effects of different salinity levels on the dry matter ratios of aboveground and underground parts of two *Brassica napus* varieties, CH336 (semi-winter type) and Brutor (spring type), measured at early flowering stage in pot cultures. Bars indicate standard deviation (SD) of the means.

The above- and underground dry matter ratios were also calculated for the seedlings at the early flowering stage under the treatment of six different salinity levels (Figure 2). In both varieties, the underground dry matter ratios first increased, reaching a relatively higher level at 50 mmol L^−1^ NaCl, then decreased along with the increase in salt concentration. However, the aboveground dry matter ratios were less affected by the different NaCl concentrations tested in the two varieties.

### 2.2. RNA-seq Analysis and Identification of Differently Expressed Genes (DEGs)

To gain insight into the mechanisms underlying the increase in seedling biomass by the lower levels (25–50 mmol L^−1^) of salinity treatments, messenger RNA (mRNA) sequencing (RNA-seq) of the shoot apical meristem (SAM) samples from the six-leaf-aged plants under the treatments of 0 mmol L^−1^ (control, CK), 25 mmol L^−1^ (low salt stress, LS), and 180 mmol L^−1^ (high salt stress, HS) NaCl treatments was performed in both varieties, CH336 and Brutor. The effective data for each sample were 6.57~7.12 G, the Q30 base rate was 94.44~94.56%, and the average GC content was 48.59%. By mapping the reads to the *B. napus* reference genome of Darmor-*bzh* (https://www.genoscope.cns.fr/brassicanapus/, accessed on 1 October 2022), the genome alignment of each sample was obtained, and the alignment rate distribution was 93.69~94.48%.

**Figure 3:**
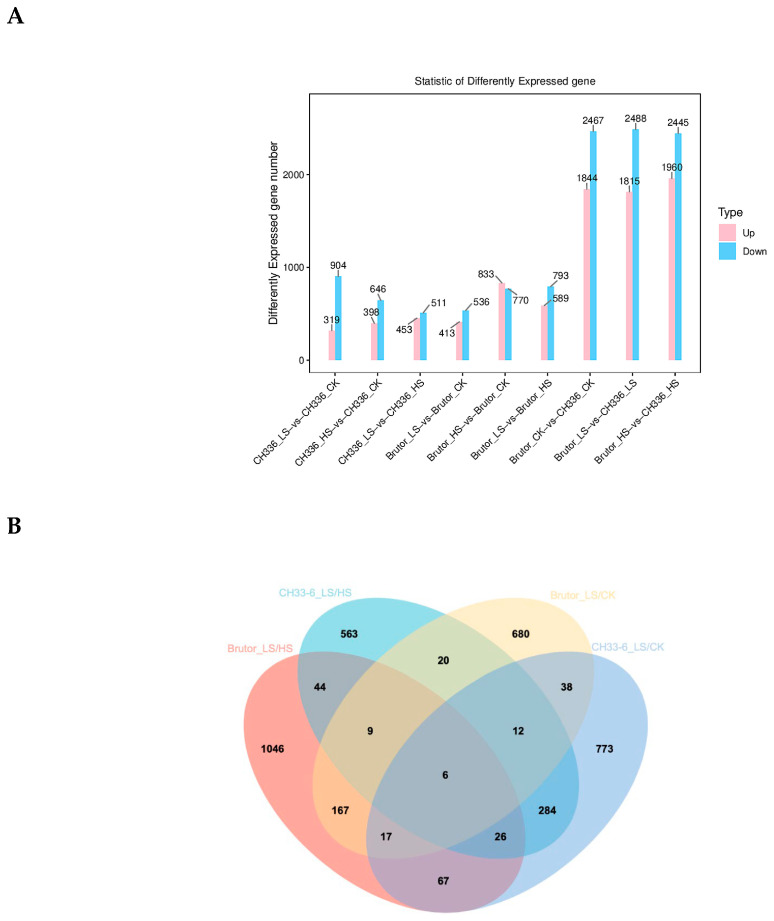
Statistical charts of the differentially expressed genes (DEGs) in two *Brassica napus* varieties (CH336 and Brutor) under control (CK, 0 mmol L^− 1^ NaCl), low salt (LS, 25 mmol L^− 1^ NaCl), and high salt (HS, 180 mmol L^− 1^ NaCl) stress conditions. (**A**). The column charts of DEGs in each comparison group. The pink and light blue colors indicate the up- and down-regulated DEGs, respectively. The numbers in the column refer to the DEG amounts in each comparison group. (**B**). Venn diagram showing the number of unique and common DEGs in both varieties.

**Figure 4:**
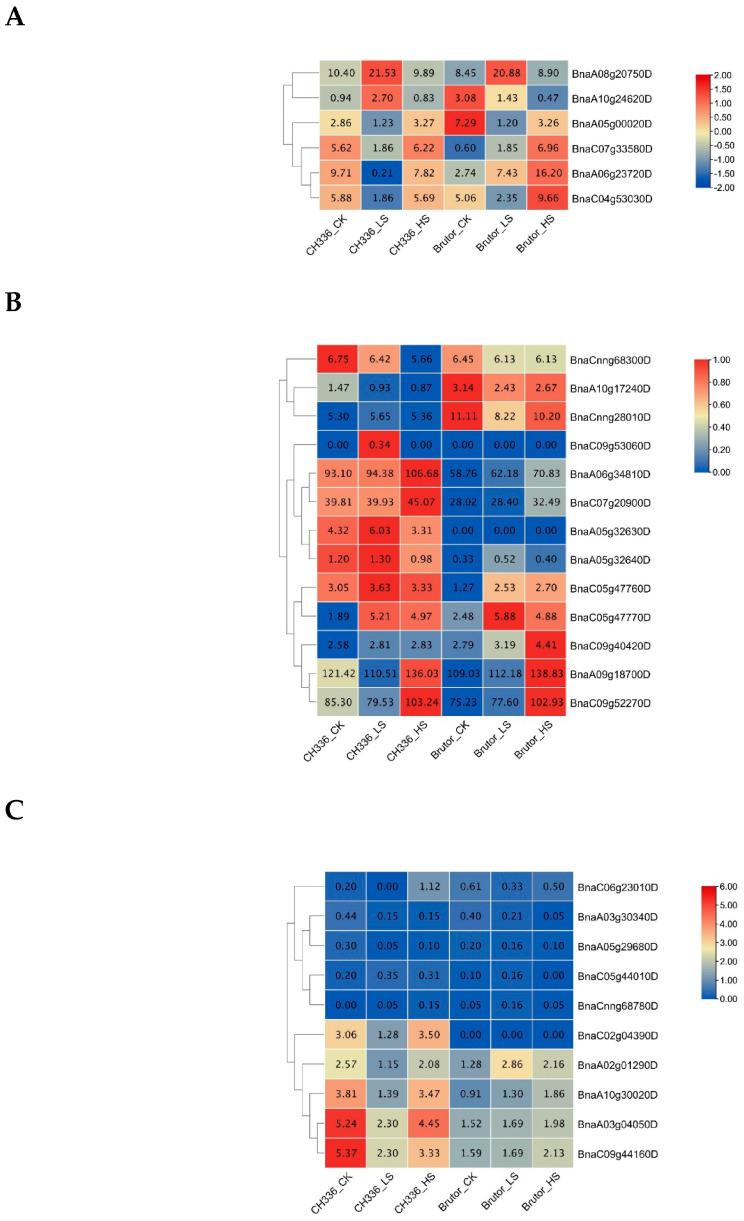
FPKM(−normalized heatmaps of some candidate differentially expressed genes (DEGs) potentially involved in stimulating the seedling growth under moderate salinity stress in *Brassica napus*. (**A**). Six DEGs commonly shared between LS/CK and LS/HS and with similar expression patterns in two oilseed rape varieties (CH336 and Brutor). (**B**). 13 *B. napus DELLA* family genes. (**C**). 10 *B. napus MYB46* genes.

The differences in gene expression levels between the samples of different treatments were then examined in the two varieties to identify the candidate genes that may be involved in the increase in seedling biomass by moderate salinity stress. The differentially expressed genes (DEGs) were identified between the different treatments in the two varieties. The number of identified DEGs was 1223 (319 up- and 904 down-regulated) between CH336_LS and CH336_CK, 1044 (398 up- and 646 down-regulated) between CH336_HS and CH336_CK, 964 (453 up- and 511 down-regulated) between CH336_LS and CH336_HS, 949 (413 up- and 536 down-regulated) between Brutor_LS and Brutor_CK, 1603 (833 up- and 770 down-regulated) between Brutor_HS and Brutor_CK, and 1382 (589 up- and 793 down-regulated) between Brutor_LS and Brutor_HS. (Figure 3A). The numbers of DEGs identified between the two varieties were four times higher than between the treatments: 4311 (1844 up- and 2467 down-regulated) between Brutor_CK and CH336_CK, 4303 (1815 up- and 2488 down-regulated) between Brutor_LS and CH336_LS, and 4405 (1960 up- and 2445 down-regulated) between Brutor_HS and CH336_HS. A total of 73 DEGs were commonly shared between CH336_LS vs CH336_CK and Brutor_LS vs Brutor_CK, of which six were also shared between CH336_LS vs CH336_HS and Brutor_LS vs Brutor_HS (Figure 3B). These six DEGs are *BnaA05g00020D*, *BnaA06g23720D, BnaA08g20750D, BnaA10g24620D, BnaC04g53030D,* and *BnaC07g33580D*. Their expression patterns are summarized in Figure 4A. Among these six DEGs, three showed similar expression patterns in the two varieties, i.e., *BnaA05g00020D* (orthologous to *At2g48120*, *PAC*) and *BnaC04g53030D* (orthologous to *At2g43570*, *CHI*) were remarkably down-regulated, while *BnaA08g20750D* (orthologous to *At1g22740*, *RabG3b*) was remarkably up-regulated in the LS treatments compared to the CK and HS treatments in both varieties (Figure 4A).

Due to the importance of the *DELLA* family genes, which are widely involved in various important processes of plant growth and development [40], the relevant 13 *B. napus DELLA* genes were selected, and their expression patterns in the shoot apical meristem (SAM) tissues of the two varieties under control, LS, and HS treatments are presented in Figure 4B. Among them, *BnaA06g34810D*, *BnaA09g18700D*, *BnaC07g20900D,* and *BnaC09g52270D* are four *RGA* (*Repressor of ga1*–*3*) genes, showing significantly higher expression levels than other *DELLA* members. In both varieties, the expression levels of these four *RGA* genes were less or not affected by the LS treatments but significantly up-regulated under the HS treatments. *BnaA05g32630D* and *BnaC05g47770D* are two *GAI* (*GA insensitive*) genes and were lowly expressed compared to the four *RGA* genes. However, *BnaC05g47770D* was up-regulated by both the LS and HS treatments in both varieties, while *BnaA05g32630D* was up-regulated by the LS and down-regulated by the HS treatment in CH336, and not expressed in Brutor. The six *RGL* (*RGA-like*) genes, including two *RGL1* (*BnaCnng28010D* and *BnaCnng68300D*), two *RGL2* (*BnaA05g32640D* and *BnaC05g47760D*), and two *RGL3* (*BnaA10g17240D* and *BnaC09g40420D*), were all lowly expressed compared to the four *RGA* genes and globally less affected by the LS and HS treatments compared to the controls. *BnaC09g53060D* was not expressed, because it is a pseudogene containing only a partial sequence of an *RGA* gene in the *B. napus* reference genome.

### 2.3. GO and KEGG Enrichment Analyses of DEGs

To investigate the molecular mechanism of the increase in seedling biomass under moderate salt stress compared with no salt stress (control), Gene Ontology (GO) and Kyoto Encyclopedia of Genes and Genomes (KEGG) enrichment analyses were performed on the DEGs identified between the LS treatments and CK controls in the two varieties. A total of 2172 DEGs were identified between the LS treatments and CK controls by combining the data from the two varieties, of which 73 DEGs were commonly shared between the two varieties (Figure 3B). They were named the TOTAL_LS/CK group and the COMMON_LS/CK group, respectively.

**Figure 5:**
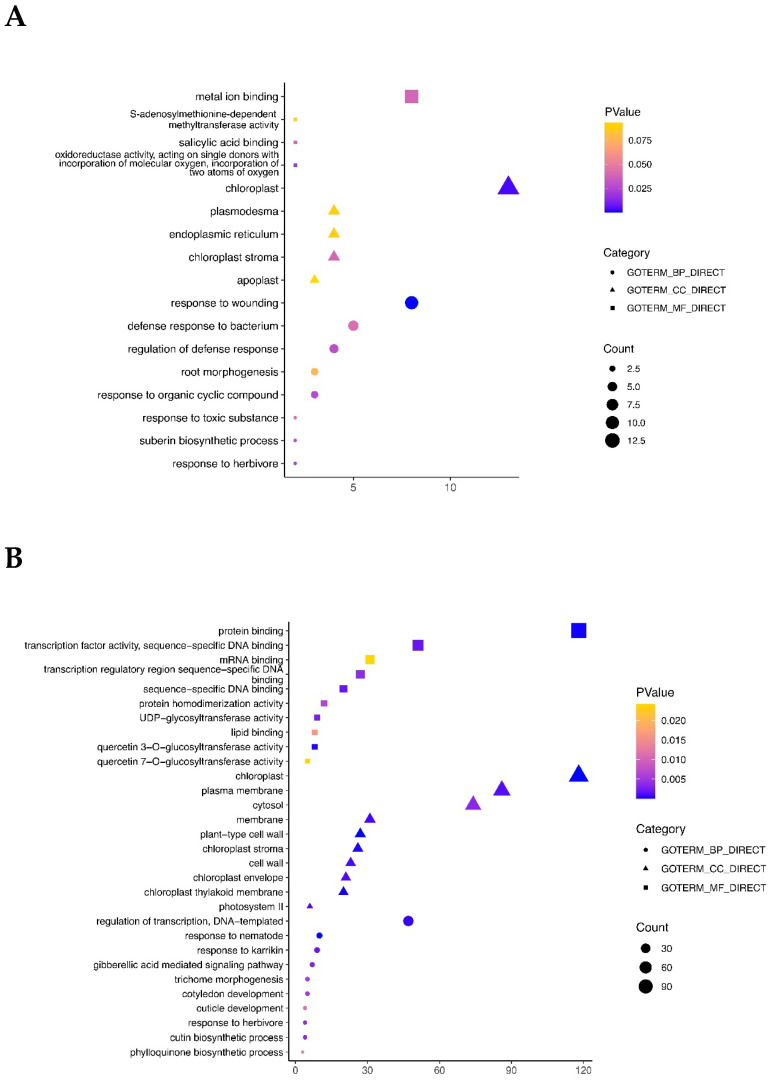

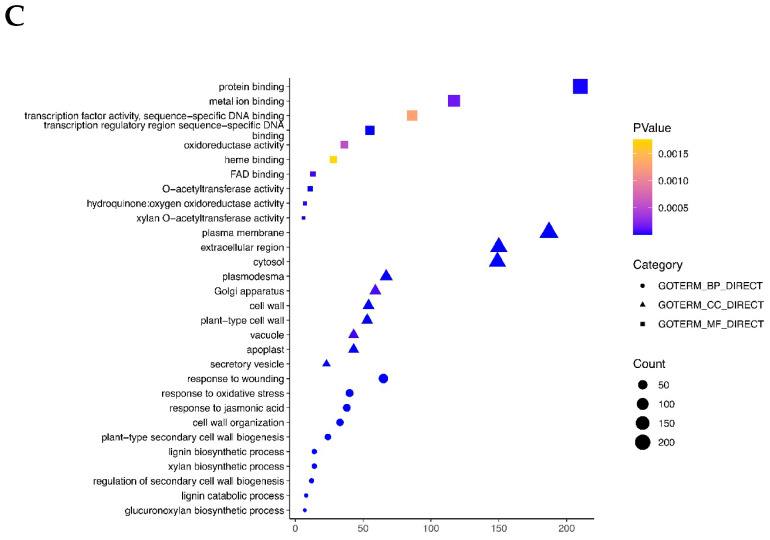
GO enrichment analysis of differentially expressed genes (DEGs) identified between low salt (LS) treatment and no salt control (CK) in two *Brassica napus* varieties (CH336 and Brutor). (**A**). GO terms of DEGs in the COMMON_LS/CK group. (**B**). GO terms of up-regulated DEGs in the TOTAL_LS/CK group. (**C**). GO terms of down-regulated DEGs in the TOTAL_LS/CK group. The square, triangle, and circle indicate the molecular function (MF), cellular component (CC), and biological process (BP) GO categories, respectively. The pattern size means the number of DEGs enriched in each term. The different pattern colors refers to different *p*-values of for the enriched GO terms.

A total of 17 GO terms were enriched in the COMMON_LS/CK group, 110 GO terms in the up-regulated DEGs (by the LS treatment) of the TOTAL_LS/CK group, and 153 GO terms in the down-regulated DEGs (by the LS treatment) of the TOTAL_LS/CK group. The top 10 GO terms of these three categories are presented in Figure 5. In the COMMON_LS/CK group, the terms such as metal ion binding (biological process, BP), chloroplast (cellular component, CC), and response to wounding (molecular function, MF) were significantly enriched (Figure 5A). In the up-regulated DEGs in the TOTAL_LS/CK group, the terms such as protein binding, transcription factor activity, sequence-specific DNA binding (MF), chloroplast, plasma, cytosol, and chloroplast-related terms such as chloroplast stroma, chloroplast envelope and chloroplast thylakoid membrane (CC), regulation of transcription, and DNA-templated (BP) were highly enriched (Figure 5B). In the down-regulated DEGs of the TOTAL_LS/CK group, the terms such as protein binding and metal ion binding (MF), plasma membrane, extracellular region and cytosol (CC), response to wounding, plant−type secondary cell wall biogenesis, the lignin biosynthetic process, and the xylan biosynthetic process (BP) were enriched (Figure 5C).

**Figure 6:**
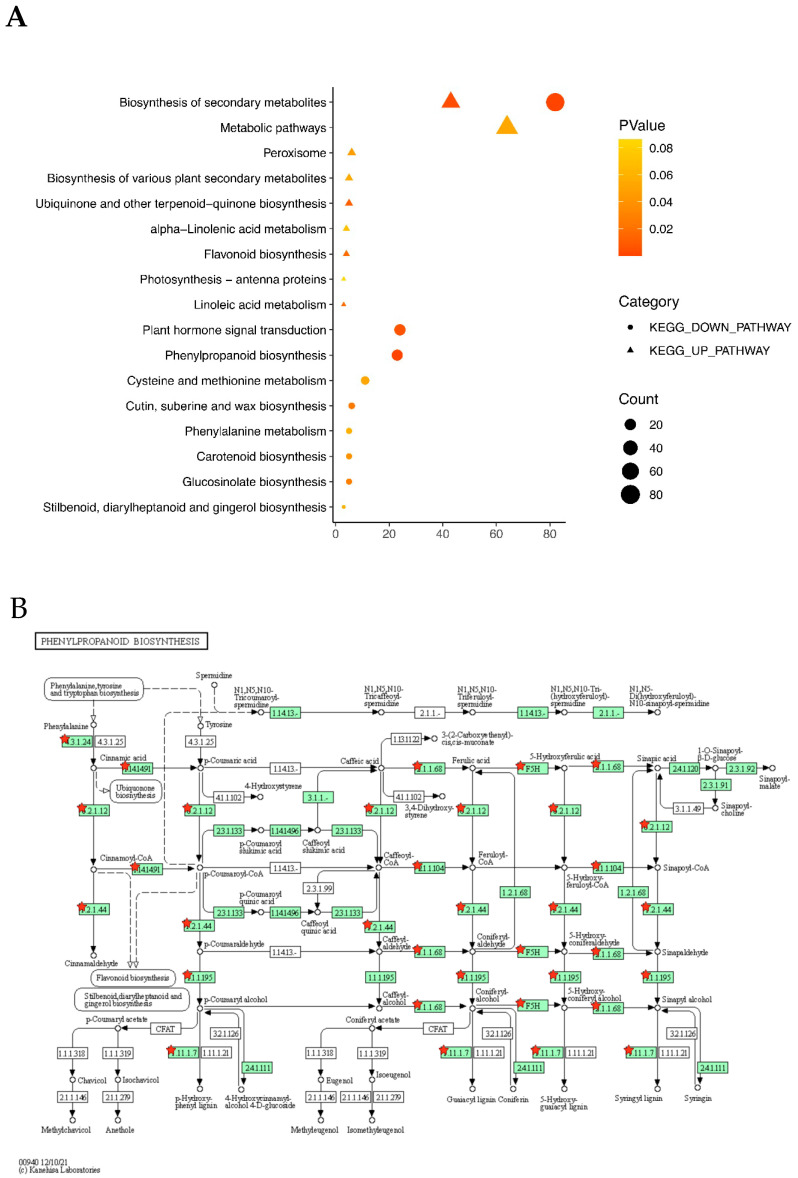
KEGG pathway enrichment analysis of differentially expressed genes (DEGs) identified between low salt (LS) treatment and no salt control (CK) in two *Brassica napus* varieties (CH336 and Brutor). (**A**). KEGG pathways of DEGs in TOTAL_LS/CK group. The triangle and circle indicate the up- and down-regulated KEGG pathways, respectively. The pattern size means the number of DEGs enriched in each pathway. The different pattern colors refers to different *p*-values for the enriched pathways. (**B**). The phenylpropanoid biosynthesis pathway. The DEGs involved in the pathway are marked by red stars.

One KEGG pathway was enriched in the COMMON_LS/CK group, nine KEGG pathways were enriched in the up-regulated DEGs (by the LS treatment) of the TOTAL_LS/CK group, and nine other KEGG pathways were enriched in the down-regulated DEGs (by the LS treatment) of the TOTAL_LS/CK group (Figure 6). The biosynthesis of the secondary metabolites pathway was enriched in all of the three above groups. For the TOTAL_LS/CK group, the number of DEGs in the down-regulated pathways was much higher than that in the up-regulated pathways (Figure 6A). The plant hormone signal transduction pathway and phenylpropanoid biosynthesis pathway were significantly enriched in the down-regulated KEGG pathways (Figure 6A). In the phenylpropanoid biosynthesis pathway, many DEGs were down-regulated by the LS treatment (Figure 6B), among which the two *PER49* genes (*BnaA01g01280D* and *BnaAnng19770D*, 1.1.1.68 in Figure 6B) were significantly down-regulated by threefold in both varieties. Lignin biosynthesis is one of the most important branches in the phenylpropanoid biosynthesis pathway [41,42]. Owing to the pivotal role of the MYB46 transcription factor in lignin deposition [43], the expression patterns of 10 *B. napus MYB46* genes under different salt stress treatments were also examined in this study (Figure 4C). Eight out of the ten *MYB46* genes showed a down-regulation of expression by the LS treatment in the semi-winter type variety, CH336. However, their expression patterns were differentin the spring type variety, Brutor, where the *MYB46* genes were lower expressed and less affected by the LS treatment.

## 3. Discussion

In this study, we first tested the effects of different concentrations of a NaCl solution on the seedling growth performance of two oilseed rape varieties, CH336 and Brutor, representing two different ecotypes (semi-winter type and spring type) in *B. napus*. We found that the moderate salt concentrations of 25 and 50 mmol L^−1^ can stimulate the seedling growth by a significant increase of 10~20% (compared to controls) in both above- and underground biomasses, as estimated at the early flowering stage, although the degree of this stimulating effect varied between the two varieties (Table 1). A further increase in the salinity stress level can inhibit plant growth, with significantly lower above- and underground biomasses. This result was in agreement with several previous studies on different plants [19,39,44]. Wassan et al. [39] analyzed a panel of 228 *B. napus* accessions (197 semi-winter types, 9 spring types) and found that a 50 mM NaCl treatment increased the average shoot dry weight (by ~30%) and average root length (by ~2%) of the seven-day-old seedlings compared to the controls. Geng et al. [19] reported that low levels of neutral salt (NaCl and Na_2_SO_4_ at 1:1, Na^+^ 25 mM) or alkaline salt (Na_2_CO_3_, Na^+^ 25 mM) treatments significantly enhanced the total biomass, leaf area, and photosynthesis indicators in 25-day-old seedlings of a sugar beet variety. Rodríguez-Hernández and Garmendia [44] reported that a 100 mmol L^–1^ NaCl treatment induced a slight increase in shoot dry weight and enhanced the leaf area in glacier lettuce (*Mesembryanthemum crystallinum* L.), an annual halophyte highly tolerant to salinity. However, the treatment of 100 mmol L^−1^ (or higher) NaCl inhibited seedling growth, as observed in our study as well as in that of Yong et al. on 85 accessions of oilseed rape [34]. In addition, a high level of neutral salt (NaCl:Na_2_SO_4_ at 1:1, Na^+^ 100 mM) significantly inhibited seedling growth and photosynthesis in sugar beet [19]. These results indicate that the stimulating of seedling growth by moderate salinity stress may be a general phenomenon for most plants, although the optimal salt concentration may be varied according to the plant species or cultivated variety. Future studies should examine if the seed yield and quality of *B. napus* are also improved by moderate salinity stress, and if there exist some oilseed rape varieties more appropriate than others to be cultivated on moderate salinity soils with enhanced seed yield and quality.

The specific leaf weight (SLW) is an index referring to the fresh or dry weight of leaves per unit leaf area, which can reflect the thickness and quality of leaves and is related to chlorophyll content, photosynthetic function, and plant development [45]. In most plants, it is positively related to water-use efficiency (WUE), and maximum light and capacity. In this study, the SLW showed a trend of increasing first and then decreasing with the increment of salt concentration (Table 1). Under low salinity treatments, the SLW increased in both varieties, which was consistent with the results of the GO enrichment analysis, which showed that the terms such as chloroplast, chloroplast envelope, and chloroplast thyroid membrane were significantly enriched in the cellular component (CC) category of up-regulated DEGs (Figure 5). Possibly, plants distribute more organic matter to photosynthesis-related thylakoids and RuBP carboxylase due to salinity [46], leading to a stronger photosynthetic capacity in leaves and a higher accumulation of photosynthetic products. With further increases in salinity, the SLW continued to rise, but the overall dry weight of the plant decreased, indicating the growth and development of oilseed rape had been influenced by salt stress, with various physical and chemical metabolic processes being weakened. The increase in the SLW may be due to the physiological drought and lower water content of the leaves induced by salt stress. Meanwhile, the stronger salt stress results in allocating more biomass to the cell wall and accumulating more photosynthetic products as compensation for normal growth and development [47].

In our RNA-seq analysis, the numbers of DEGs identified between the two varieties were four times higher than those between any two treatments in a single variety (Figure 3A). This implies that the genetic background is very different between the semi-winter-type and spring-type oilseed rape varieties. In two DEGs, *BnaA05g00020D* and *BnaC04g53030D*, the expression levels were remarkably down-regulated by the low-salinity (LS) treatment in both varieties compared to the control (CK) and the high-salinity (HS) treatments (Figure 4A). *BnaA05g00020D* (orthologous to *At2g48120, PAC*, *pale cress*) is considered to be essential for the early development of chloroplasts, plays an important role in the formation of chloroplast tissue and leaf morphogenesis, and participates in the response of cells to light stimulation [48]. *BnaC04g53030D* (orthologous to *At2G43570, CHI*) encodes a putative basic chitinase, a pathogenesis-related (PR) protein that is considered to be involved in the defense against external bio- and abiotic stresses, as well as in the senescence of photosynthetic tissues in plants [49,50]. On the other hand, *BnaA08g20750D* (orthologous to *At1G22740*, *RabG3b*) showed to be remarkably up-regulated by the low-salinity (LS) treatment in both varieties compared to the control (CK) and high-salinity (HS) treatments. It encodes Rab GTPase RabG3b, which contributes to tracheary element differentiation in the *Arabidopsis xylem* as a component of autophagy [49]. The tracheary elements of the xylem serve as the water-conducting vessels of the plant vascular system, with secondary cell wall thickening and cell death, during which the cell contents are completely removed [51]. Interestingly, a previous study showed that overexpression of constitutively active *Arabidopsis* RabG3b could promote xylem development and increases stem growth in transgenic poplars [52]. So, *BnaA05g00020D*, *BnaC04g53030D,* and *BnaA08g20750D* are good candidate genes for being involved in stimulating oilseed rape seedling growth in low- or moderate-salinity stress. These genes may engage in the regulation of source-sink in oilseed rape, help redirect the energy to the formation of biomass, and reduce the energy loss of secondary metabolic biosynthesis induced by salt stress.

The *DELLA* family genes are widely involved in various important growth and development processes of plants and play an important role in plant stress response through the gibberellin (GA) signaling pathway [40,53]. DELLA proteins are considered master negative regulators of GA signaling. Under stress conditions, DELLA enhances the expression of proteins involved in cell cycle inhibition, which is considered an acclimatization strategy by most plants to reserve resources for survival [54,55]. The *A. thaliana* genome contains five *DELLA* genes, named *GA-Insensitive* (*GAI*), *Repressor of ga1*−*3* (*RGA*), *RGA-Like1* (*RGL1*), *RGA-Like2* (*RGL2*), and *RGA-Like3* (*RGL3*). *AtGAI* and *AtRGA* have partially redundant functions in maintaining the repressive state of the GA-signaling pathway and repressing the plant vegetative growth, but RGA plays a more dominant role than GAI [56,57]. *AtRGL1* positively regulated age-triggered leaf senescence [58] and negatively regulated embryo sac development [59]. *AtRGL2* repressed seed germination [60,61], and *AtRGL3* positively regulated the jasmonic acid (JA)- and salicylic acid (SA)-mediated response against pathogen infections [62]. In this study, the four *B. napus RGA* genes (*BnaA06g34810D*, *BnaA09g18700D*, *BnaC07g20900D*, and *BnaC09g52270D*) were revealed to be highly expressed in *B. napus* shoot apical meristem (SAM) tissues compared to other *DELLA* genes (Figure 4B). Their expression levels were not affected by LS but significantly increased by HS treatments, indicating that these *RGA* genes may have contributed to the reduction of oilseed rape seedling growth (through the GA signaling pathway) under the HS stress observed in this study (Figure 1, Table 1).

Our GO enrichment analysis showed that many photosynthesis-related GO terms were significantly enriched in the up-regulated DEGs under LS treatment in the two *B. napus* varieties, including the chloroplast, chloroplast stroma, chloroplast envelope, and chloroplast thylakoid membrane terms (Figure 5). The genes in these terms that promote photosynthesis efficiency were enhanced to alleviate salt stress. Down-regulation of DEGs of various biosynthetic process (BP) terms, including plant-type secondary cell wall biogenesis, lignin biosynthetic process, xylan biosynthetic process, regulation of secondary cell wall biogenesis, lignin catabolic process, and glucuronoxylan biosynthetic process, reduced energy loss from the biosynthesis of the secondary metabolites.

Our KEGG enrichment analysis also showed that a secondary metabolic pathway, the phenylpropanoid biosynthesis pathway, was significantly enriched in the down-regulated DEGs of the LS/CK group (Figure 6). As the main metabolic pool of photosynthetically fixed carbon, phenylpropanoid metabolism consumes a large amount of substance and energy in plants. It can metabolize 30~40% of the photosynthetic fixed organic carbon into downstream products such as flavonoids, cellulose, or hemicellulose [41], and the precursors of lignin, lignans, and tannins [63]. Such allocation among different branches of phenylpropanoid metabolism is achieved via the regulation of metabolic flux redirection, with extraordinary complexity in response to environmental stimuli [41,64]. Lignin is one of the end products of phenylpropanoid biosynthesis and the secondary cell wall components. MYB transcription factors (TF) are required for secondary cell wall biosynthesis, including lignin deposition [63,65]. For example, *MYB221* negatively regulates lignin biosynthesis. In previous studies, researchers expressed both *PdGA20ox1* and *PtrMYB221* in *Populus* wood-forming tissue to reduce the lignin content, thus improving the woody biomass by twofold [43]. Among MYB TF, MYB46 plays a pivotal role and positively regulates the lignin biosynthesis process [41,66]. In this study, eight out of the ten *MYB46* genes showed a down-regulation of expression by the LS treatment in the semi-winter-type variety CH336 (Figure 4C). This may reduce the lignin synthesis, save more energy for primary metabolism to avoid excessive secondary metabolic energy and material consumption, and thus promote the seedling biomass in the semi-winter-type variety, CH336. However, such a mechanism seems not plausible in the spring-type variety, Buttor.

## 4. Materials and Methods

### 4.1. Plant Materials and Growth Conditions

Pot culture experiments were conducted in three different screen houses at the Jinhua Academy of Agricultural Sciences, Zhejiang Province from late October 2021. Two *B. napus* varieties, Brutor and CH336, were selected as the tested plant materials for the study, due to their seed-coat thickness differences, as well as their similar flowering dates and suitability for growing in local areas. Brutor is a spring-type variety. originated from France, with a thin seed coat and a flowering time of 156 days after sowing (DAS) to first flowering, while CH336 is a semi-winter-type variety, originated from China, with a thick seed coat and a flowering time of 153 DAS to early flowering. The sowing was performed with plugs at first; then, the seedlings were transplanted at the three-leaf stage to plastic permeable pots (32.5 cm × 25 cm), with holes at the bottom, and filled with 5 kg planting substrate mixed with 10 g of compound fertilizer of potassium sulfate and 10 g of urea per pot. An additional 10 g of boron fertilizer was applied per pot just before bolting and flowering.

### 4.2. Salt Solution Treatments

The salinity treatments were performed one week after the transplanting of the seedlings to plastic pots by adding, each time, 700 mL of appropriate saline solution to each pot at a regular interval of every two weeks, until the early flowering stage. Six NaCl concentrations were used to create different levels of salinity stress, including 0 mmol L^−1^ (water control, CK), 25, 50, and 75 mmol L^−1^ (low salt stress, LS), 120 mmol L^−1^ (medium salt stress, MS), and 180 mmol L^−1^ (high salt stress, HS). Each treatment consisted of 12 pots (one plant per pot). The six treatments were repeated in three different screen houses.

### 4.3. Measurements of Plant Growth Parameters

At the six-leaf stage, the plant height was measured for the plants from each treatment. At the early flowering stage, three plants (from three different pots) of each salt treatment were randomly selected to estimate their biomass. The aboveground and the underground parts of each collected plant were separated and weighed individually to estimate the aboveground and underground fresh biomass. For the underground part, the roots were rinsed with water, wiped dry, and weighed for fresh biomass.

To estimate the dry matter ratio, the two parts of each plant were dried in an oven for 20 min at 105 °C, followed by 24 h at 60 °C, and then weighed to estimate their dry biomass. The dry matter ratio was calculated using the following formula:

Dry matter ratio (%) = (dry weight/fresh weight) × 100(1)

The specific leaf weight (SLW), also known as leaf dry weight per unit leaf area, reflects the ability of plant leaves to obtain light resources and, thus, can be used to assess the photosynthesis ability of plants. To estimate the specific leaf weight (SLW), fresh leaves (avoiding the main veins) were cut into pieces of 2 × 2 = 4 cm^2^, dried in an oven, as above, and then weighed. The calculation formula is as follows:

Specific leaf weight (g/cm^2^) = dry leaf weight/leaf area (2)

In each case, the samples were randomly collected, and each treatment was repeated three times to calculate the average value.

### 4.4. RNA Isolation

The shoot apical meristem (SAM) samples were collected from the six-leaf-aged seedlings of the two varieties under the treatment of 0 mmol L^−1^ (control, CK), 25 mmol L^−1^ (Low salt stress, LS), and 180 mmol L^−1^ (High salt stress, HS) NaCl solutions, named as Brutor_CK, Brutor_LS, Brutor_HS, CH336_CK, CH336_LS, and CH336_HS, for the following transcriptomic analysis. The SAM tissues were sampled and frozen in liquid nitrogen and stored at −80 °C. Total RNA was extracted using the RNAprep Pure Plant Kit from Tiangen Biotech Co., Ltd. (Beijing, China) according to the manufacturer’s protocol. The RNA purity and quantification were evaluated using the NanoDrop 2000 spectrophotometer (Thermo Scientific, USA). The RNA integrity was assessed using the Agilent 2100 Bioanalyzer (Agilent Technologies, Santa Clara, CA, USA). The samples with RNA Integrity Number (RIN) ≥ 7 were subjected to the subsequent analysis.

### 4.5. Construction of cDNA Libraries and RNA Sequencing

The cDNA libraries were constructed using the TruSeq Stranded mRNA LT Sample Prep Kit (Illumina, San Diego, CA, USA) according to the manufacturer’s instructions. The transcriptome sequencing and analysis were conducted by OE Biotech Co., Ltd. (Shanghai, China). The cDNA libraries were sequenced on an Illumina HiSeq X Ten platform, and 150 bp paired-end reads were generated.

### 4.6. Identification of Differentially Expressed Genes (DEGs) and Functional Analysis

The raw reads of the FASTQ format were first processed using Trimmomatic 0.36 software [67], and the low-quality reads were removed to obtain the clean reads. The clean reads were mapped onto the *B. napus* reference genome of Darmor-*bzh* (*Brassica napus* v4.1, https://www.genoscope.cns.fr/brassicanapus/, accessed on 1 October 2022) by Hisat2 [68]. The FPKM (fragments per kilobase of exon model per million mapped reads) value of each gene was calculated using Cufflinks software [69], and the read counts of each gene were obtained by HTSeq-count [70]. The differential expression analysis was performed using the DESeq (2012) R package, and the *p*-value < 0.05 and |log_2_ Foldchange| > 2 were set as the thresholds by default for significant differential expression.

The Gene Ontology (GO) enrichment and Kyoto Encyclopedia of Genes and Genomes (KEGG) [71] pathway enrichment analysis of the DEGs were performed using R, based on the hypergeometric distribution, to investigate their involved function and biological pathway. GO analysis contains three categories: biological process (BP), cellular component (CC), and molecular function (MF). A combined analysis of DEGs between the LS and CK (LS/CK) in the two varieties was performed to identify the common and total DEGs of the two LS/CK groups, named COMMON_LS/CK and TOTAL_LS/CK, respectively, in the two varieties. The top 10 GO terms in each category and all the KEGG pathways were displayed.

### 4.7. Statistical Analyses

The statistical analyses were performed via GraphPad Prism software version 9 (https://www.graphpad.com/, accessed on 1 March 2023). The seedling growth data were first checked for normality using the Shapiro–Wilk test, and for homogeneity of variance using the *F*-test. The one-way analysis of variance (ANOVA) was then performed for evaluation of the significant difference between the means, followed by Tukey’s test (*p* < 0.05) for multiple comparisons. The values in Table 1 are represented as the mean ± standard errors (SE) of the means of three independent experiments, while, in Figure 3 (dry matter ratio values), the values are represented by the mean ± standard deviation (SD).

## 5. Conclusions

In this study, we first tested the effects of different concentrations of a NaCl solution on the seedling growth performance of two oilseed rape varieties (a semi-winter type and a spring type) and found that the moderate salt concentrations (25 and 50 mmol L^−1^ NaCl) can stimulate the seedling growth by a significant increase (10~20%, compared to the controls) in both the above- and underground biomasses, as estimated at the early flowering stage. RNA-seq analyses of shoot apical meristem (SAM) tissues from six-leaf-aged seedlings under the control (CK), low (LS, 25 mmol L^−1^ NaCl), and high (HS, 180 mmol L^−1^ NaCl) salinity treatments allowed us to identify 1223 DEGs (319 up- and 904 down-regulated) between the CH336_LS/CK, 1044 (398 up- and 646 down-regulated) between CH336_HS/CK, 964 (453 up- and 511 down-regulated) between CH336_LS/HS, 949 (413 up- and 536 down-regulated) between Brutor_LS/CK, 1603 (833 up- and 770 down-regulated) between Brutor_HS/CK, and 1382 (589 up- and 793 down-regulated) between Brutor_LS/HS. The GO and KEGG enrichment analyses of these DEGs demonstrated that such a stimulating effect of seedling growth by low salinity stress may be caused by a more efficient capacity for photosynthesis as compensation, accompanied by a reduced energy loss for the biosynthesis of the secondary metabolites and redirecting of energy to biomass formation.

## Figures and Tables

**Table 1:** Effects of different salinity levels on the seedling growth parameters of two *Brassica napus* varieties, CH336 (semi-winter type) and Brutor (spring type), in pot cultures.

Variety	Salinity (mmol L^−1^)	Plant Height at Six-Leaf Stage (cm)	Aboveground Fresh Weight at Early Flowering Stage (g)	Underground Fresh Weight at Early Flowering Stage (g)	Aboveground Dry Weight at Early Flowering Stage (g)	Underground Dry Weight at Early Flowering Stage (g)	Specific Leaf Weight (SLW) at Early Flowering Stage (µg/cm^2^)
CH336	0	32.7 ± 1.4 ^b^	244.39 ± 14.27 ^a^	16.23 ± 0.99 ^b^	29.47 ± 1.94 ^ab^	2.51 ± 0.26 ^b^	4.89 ± 0.37 ^a^
	25	43.7 ± 2.0 ^a^	281.75 ± 15.70 ^a^	19.37 ± 1.08 ^ab^	33.64 ± 1.56 ^a^	3.46 ± 0.30 ^ab^	4.94 ± 0.27 ^a^
	50	42.0 ± 1.2 ^a^	286.37 ± 12.88 ^a^	20.90 ± 1.40 ^a^	35.30 ± 2.05 ^a^	3.73 ± 0.25 ^a^	5.41 ± 0.10 ^a^
	75	35.1 ± 1.4 ^b^	235.81 ± 12.69 ^ab^	14.94 ± 0.70 ^bc^	24.27 ± 1.08 ^b^	2.53 ± 0.14 ^b^	5.13 ± 0.91 ^a^
	120	34.4 ± 0.8 ^b^	185.27 ± 8.88 ^b^	14.54 ± 0.98 ^bc^	23.22 ± 1.75 ^bc^	2.83 ± 0.27 ^ab^	5.32 ± 0.47 ^a^
	180	24.0 ± 1.6 ^c^	133.53 ± 5.10 ^c^	10.75 ± 0.87 ^c^	16.33 ± 1.29 ^c^	1.82 ± 0.30 ^b^	5.16 ± 0.32 ^a^
Brutor	0	44.9 ± 1.2 ^bc^	646.58 ± 32.05 ^ab^	46.21 ± 2.86 ^b^	69.02 ± 3.44 ^ab^	8.92 ± 0.77 ^b^	3.76 ± 0.53 ^a^
	25	60.2 ± 2.6 ^a^	700.47 ± 45.55 ^a^	59.51 ± 4.63 ^a^	74.96 ± 4.49 ^ab^	12.44 ± 1.24 ^ab^	3.99 ± 0.40 ^a^
	50	47.9 ± 1.2 ^b^	691.86 ± 44.52 ^a^	66.16 ± 3.34 ^a^	82.90 ± 5.34 ^a^	14.70 ± 0.82 ^a^	4.14 ± 0.63 ^a^
	75	46.6 ± 1.5 ^bc^	612.30 ± 31.61 ^ab^	56.14 ± 2.41 ^ab^	71.09 ± 3.77 ^ab^	13.11 ± 0.65 ^ab^	4.49 ± 0.80 ^a^
	120	39.6 ± 1.4 ^c^	545.23 ± 21.52 ^b^	43.67 ± 2.82 ^b^	64.09 ± 4.92 ^b^	10.15 ± 0.79 ^b^	4.46 ± 0.12 ^a^
	180	38.3 ± 0.7 ^c^	436.96 ± 13.67 ^b^	28.13 ± 0.95 ^c^	44.56 ± 1.54 ^c^	5.17 ± 0.35 ^c^	3.90 ± 0.36 ^a^

Notes: Means ± SE (standard errors of means) were compared with Tukey’s test. Within each column, values followed by a common letter are not significantly different (*p* < 0.05).

## Data Availability

The data supporting the findings of this study are included in this article.

## References

[1] Singh A. (2022). Soil Salinity: A Global Threat to Sustainable Development. Soil Use Manag..

[2] Haj-Amor Z., Araya T., Kim D.G., Bouri S., Lee J., Ghiloufi W., Yang Y., Kang H., Jhariya M.K., Banerjee A. (2022). Soil Salinity and Its Associated Effects on Soil Microorganisms, Greenhouse Gas Emissions, Crop Yield, Biodiversity and Desertification: A Review. Sci. Total Environ..

[3] Hopmans J.W., Qureshi A.S., Kisekka I., Munns R., Taleisnik E. (2021). Critical Knowledge Gaps and Research Priorities in Global Soil Salinity. Adv. Agron..

[4] Singh A. (2018). Alternative Management Options for Irrigation-Induced Salinization and Waterlogging Under Different Climatic Conditions. Ecol. Indic..

[5] Arif Y., Singh P., Siddiqui H., Bajguz A., Hayat S. (2020). Salinity Induced Physiological and Biochemical Changes in Plants: An Omic Approach Towards Salt Stress Tolerance. Plant Physiol. Biochem..

[6] Hameed A., Ahmed M.Z., Hussain T., Aziz I., Ahmad N., Gul B., Nielsen B.L. (2021). Effects of Salinity Stress on Chloroplast Structure and Function. Cells.

[7] Zörb C., Geilfus C.M., Dietz K.J. (2019). Salinity and Crop Yield. Plant Biol..

[8] Munns R., Tester M. (2008). Mechanisms of salinity tolerance. Annu. Rev. Plant Biol..

[9] Zhao C., Zhang H., Song C., Zhu J.K., Shabala S. (2020). Mechanisms of Plant Responses and Adaptation to Soil Salinity. Innovation.

[10] Rahman M.M., Mostofa M.G., Keya S.S., Siddiqui M.N., Ansary M.M.U., Das A.K., Rahman M.A., Tran L.S. (2021). Adaptive Mechanisms of Halophytes and Their Potential in Improving Salinity Tolerance in Plants. Int. J. Mol. Sci..

[11] Chourasia K.N., More S.J., Kumar A., Kumar D., Singh B., Bhardwaj V., Kumar A., Das S.K., Singh R.K., Zinta G. (2022). Salinity Responses and Tolerance Mechanisms in Underground Vegetable Crops: An Integrative Review. Planta.

[12] Bartels D., Dinakar C. (2013). Balancing Salinity Stress Responses in Halophytes and Non-Halophytes: A Comparison between *Thellungiella* and *Arabidopsis thaliana*. Funct. Plant Biol..

[13] Prerostova S., Dobrev P.I., Gaudinova A., Hosek P., Soudek P., Knirsch V., Vankova R. (2017). Hormonal Dynamics During Salt Stress Responses of Salt-Sensitive *Arabidopsis thaliana* and Salt-Tolerant *Thellungiella salsuginea*. Plant Sci..

[14] Muthuramalingam P., Jeyasri R., Rakkammal K., Satish L., Shamili S., Karthikeyan A., Valliammai A., Priya A., Selvaraj A., Gowri P. (2022). Multi-Omics and Integrative Approach Towards Understanding Salinity Tolerance in Rice: A Review. Biology.

[15] Abuslima E., Kanbar A., Raorane M.L., Eiche E., Junker B.H., Hause B., Riemann M., Nick P. (2022). Gain Time to Adapt: How Sorghum Acquires Tolerance to Salinity. Front. Plant Sci..

[16] Trono D., Pecchioni N. (2022). Candidate Genes Associated with Abiotic Stress Response in Plants as Tools to Engineer Tolerance to Drought, Salinity and Extreme Temperatures in Wheat: An Overview. Plants.

[17] Mwando E., Angessa T.T., Han Y., Li C. (2020). Salinity Tolerance in Barley during Germination- Homologs and Potential Genes. J. Zhejiang Univ. Scien. B.

[18] Maryum Z., Luqman T., Nadeem S., Khan S.M.U.D., Wang B., Ditta A., Khan M.K.R. (2022). An Overview of Salinity Stress, Mechanism of Salinity Tolerance and Strategies for Its Management in Cotton. Front. Plant Sci..

[19] Geng G., Li R., Stevanato P., Lv C., Lu Z., Yu L., Wang Y. (2020). Physiological and Transcriptome Analysis of Sugar Beet Reveals Different Mechanisms of Response to Neutral Salt and Alkaline Salt Stresses. Front. Plant Sci..

[20] Acharya B.R., Sandhu D., Dueñas C., Ferreira J.F.S., Grover K.K. (2022). Deciphering Molecular Mechanisms Involved in Salinity Tolerance in Guar (*Cyamopsis tetragonoloba* (L.) Taub.) Using Transcriptome Analyses. Plants.

[21] Gill R.A., Helal M.M.U., Tang M., Hu M., Tong C., Liu S. (2023). High-Throughput Association Mapping in *Brassica napus* L.: Methods and Applications. Methods Mol. Biol..

[22] Chalhoub B., Denoeud F., Liu S., Parkin I.A., Tang H., Wang X., Chiquet J., Belcram H., Tong C., Samans B. (2014). Early Allopolyploid Evolution in the Post-Neolithic *Brassica napus* Oilseed Genome. Science.

[23] Sun F., Fan G., Hu Q., Zhou Y., Guan M., Tong C., Li J., Du D., Qi C., Jiang L. (2017). The High-Quality Genome of *Brassica napus* Cultivar ’ZS11’ Reveals the Introgression History in Semi-Winter Morphotype. Plant J..

[24] Song J.M., Guan Z., Hu J., Guo C., Yang Z., Wang S., Liu D., Wang B., Lu S., Zhou R. (2020). Eight High-Quality Genomes Reveal Pan-Genome Architecture and Ecotype Differentiation of *Brassica napus*. Nat. Plants.

[25] Wu D., Liang Z., Yan T., Xu Y., Xuan L., Tang J., Zhou G., Lohwasser U., Hua S., Wang H. (2019). Whole-Genome Resequencing of a Worldwide Collection of Rapeseed Accessions Reveals the Genetic Basis of Ecotype Divergence. Mol. Plant.

[26] Wu J., Li F., Xu K., Gao G., Chen B., Yan G., Wang N., Qiao J., Li J., Li H. (2014). Assessing and Broadening Genetic Diversity of a Rapeseed Germplasm Collection. Breed Sci..

[27] Hu Q., Hua W., Yin Y., Zhang X., Liu L., Shi J., Zhao Y., Qin L., Chen C., Wang H. (2017). Rapeseed Research and Production in China. Crop J..

[28] Raza A. (2021). Eco-Physiological and Biochemical Responses of Rapeseed (*Brassica napus* L.) to Abiotic Stresses: Consequences and Mitigation Strategies. J. Plant Growth Regul..

[29] Shahzad B., Rehman A., Tanveer M., Wang L., Park S.K., Ali A. (2022). Salt Stress in *Brassica*: Effects, Tolerance Mechanisms, and Management. J. Plant Growth Regul..

[30] Naeini Z. (2007). Effects of Salinity Stress on the Morphology and Yield of Two Cultivars of Canola (*Brassica napus* L.). J. Agron..

[31] Arif M.R., Islam M.T., Robin A.H.K. (2019). Salinity Stress Alters Root Morphology and Root Hair Traits in *Brassica napus*. Plants.

[32] Mohamed I.A.A., Shalby N., Bai C., Qin M., Agami R.A., Jie K., Wang B., Zhou G. (2020). Stomatal and Photosynthetic Traits Are Associated with Investigating Sodium Chloride Tolerance of *Brassica Napus* L. Cultivars. Plants.

[33] Mohamed I.A.A., Shalby N., El-Badri A.M., Batool M., Wang C., Wang Z., Salah A., Rady M.M., Jie K., Wang B. (2022). RNA-Seq Analysis Revealed Key Genes Associated with Salt Tolerance in Rapeseed Germination through Carbohydrate Metabolism, Hormone, and MAPK Signaling Pathways. Ind. Crops Prod..

[34] Yong H.Y., Wang C., Bancroft I., Li F., Wu X., Kitashiba H., Nishio T. (2015). Identification of a Gene Controlling Variation in the Salt Tolerance of Rapeseed (*Brassica napus* L.). Planta.

[35] Dolatabadi N., Toorchi M. (2017). Rapeseed (*Brassica napus* L.) Genotypes Response to NaCl Salinity. J. Biodiv. Environ. Sci..

[36] Wan H., Chen L., Guo J., Li Q., Wen J., Yi B., Ma C., Tu J., Fu T., Shen J. (2017). Genome-Wide Association Study Reveals the Genetic Architecture Underlying Salt Tolerance-Related Traits in Rapeseed (*Brassica napus* L). Front. Plant Sci..

[37] Wan H., Wei Y., Qian J., Gao Y., Wen J., Yi B., Ma C., Tu J., Fu T., Shen J. (2018). Association Mapping of Salt Tolerance Traits at Germination Stage of Rapeseed (*Brassica napus* L). Euphytica.

[38] Wu H., Guo J., Wang C., Li K., Zhang X., Yang Z., Li M., Wang B. (2019). An Effective Screening Method and a Reliable Screening Trait for Salt Tolerance of *Brassica Napus* at the Germination Stage. Front. Plant Sci..

[39] Wassan G.M., Khanzada H., Zhou Q., Mason A.S., Keerio A.A., Khanzada S., Solangi A.M., Faheem M., Fu D., He H. (2021). Identification of Genetic Variation for Salt Tolerance in *Brassica napus* Using Genome-Wide Association Mapping. Mol. Genet. Genom..

[40] Davière J.M., Achard P. (2016). A pivotal role of DELLAs in regulating multiple hormone signals. Mol. Plant..

[41] Novaes E., Kirst M., Chiang V., Winter-Sederoff H., Sederoff R. (2010). Lignin and Biomass: A Negative Correlation for Wood Formation and Lignin Content in Trees. Plant Physiol..

[42] Dong N., Lin H. (2021). Contribution of Phenylpropanoid Metabolism to Plant Development and Plant–Environment Interactions. J. Integr. Plant Biol..

[43] Cho J.S., Jeon H.W., Kim M.H., Vo T.K., Kim J., Park E.J., Choi Y.I., Lee H., Han K.H., Ko J.H. (2019). Wood Forming Tissue-Specific Bicistronic Expression of *Pdga20ox1* and *Ptrmyb221* Improves Both the Quality and Quantity of Woody Biomass Production in a Hybrid Poplar. Plant Biotechnol. J..

[44] Rodríguez-Hernández M.D.C., Garmendia I. (2022). Optimum Growth and Quality of the Edible Ice Plant Under Saline Conditions. J. Sci. Food Agric..

[45] Niinemets Ü. (1999). Components of Leaf Dry Mass per Area-Thickness and Density-Alter Leaf Photosynthetic Capacity in Reverse Directions in Woody Plants. New Phytol..

[46] Cheng J.F., Dai T.B., Jiang H.Y., Pan X.Y., Cao W.X. (2012). Characterization of Leaf Carbon and Nitrogen Assimilation in Different Rice Genotypes at Jointing Stage and Their Relationships with Nitrogen Utilization Efficiency. Chin. J. Rice Sci..

[47] Rosati A., Esparza G., DeJong T.M., Pearcy R.W. (1999). Influence of Canopy Light Environment and Nitrogen Availability on Leaf Photosynthetic Characteristics and Photosynthetic Nitrogen-Use Efficiency of Field-Grown Nectarine Trees. Tree Physiol..

[48] Meurer J., Schmid L.M., Stoppel R., Leister D., Brachmann A., Manavski N. (2017). PALE CRESS Binds to Plastid Rnas and Facilitates the Biogenesis of the 50S Ribosomal Subunit. Plant J..

[49] Zhu M., Lu S., Zhuang M., Zhang Y., Lv H., Ji J., Hou X., Fang Z., Wang Y., Yang L. (2021). Genome-Wide Identification and Expression Analysis of the *Brassica Oleracea* L. Chitin-Binding Genes and Response to Pathogens Infections. Planta.

[50] Hanfrey C., Fife M., Buchanan-Wollaston V. (1996). Leaf Senescence in *Brassica napus*: Expression of Genes Encoding Pathogenesis-Related Proteins. Plant Mol. Biol..

[51] Kwon S.I., Cho H.J., Jung J.H., Yoshimoto K., Shirasu K., Park O.K. (2010). The Rab GTPase RabG3b Functions in Autophagy and Contributes to Tracheary Element Differentiation in *Arabidopsis*. Plant J..

[52] Kwon S.I., Cho H.J., Lee J.S., Jin H., Shin S.J., Kwon M., Noh E.W., Park O.K. (2011). Overexpression of Constitutively Active *Arabidopsis* Rabg3b Promotes Xylem Development in Transgenic Poplars. Plant Cell Environ..

[53] Colebrook E.H., Thomas S.G., Phillips A.L., Hedden P. (2014). The Role of Gibberellin Signalling in Plant Responses to Abiotic Stress. J. Exp. Biol..

[54] Achard P., Gusti A., Cheminant S., Alioua M., Dhondt S., Coppens F., Beemster G.T., Genschik P. (2009). Gibberellin Signaling Controls Cell Proliferation Rate in *Arabidopsis*. Curr. Biol..

[55] Li Y., Yang Y., Hu Y., Liu H., He M., Yang Z., Kong F., Liu X., Hou X. (2019). DELLA and EDS1 Form a Feedback Regulatory Module to Fine-Tune Plant Growth-Defense Tradeoff in *Arabidopsis*. Mol. Plant..

[56] Dill A., Sun T.P. (2001). Synergistic Derepression of Gibberellin Signaling by Removing RGA and GAI Function in *Arabidopsis thaliana*. Genetics.

[57] King K.E., Moritz T., Harberd N.P. (2001). Gibberellins are not Required for Normal Stem Growth in *Arabidopsis thaliana* in the Absence of GAI and RGA. Genetics.

[58] Chen L., Xiang S., Chen Y., Li D., Yu D. (2017). *Arabidopsis* WRKY45 Interacts with the DELLA Protein RGL1 to Positively Regulate Age-Triggered Leaf Senescence. Mol. Plant..

[59] Gomez M.D., Barro-Trastoy D., Fuster-Almunia C., Tornero P., Alonso J.M., Perez-Amador M.A. (2020). Gibberellin-Mediated RGA-LIKE1 Degradation Regulates Embryo Sac Development in *Arabidopsis*. J. Exp. Bot..

[60] Lee S.C., Cheng H., King K.E., Wang W.F., He Y.W., Hussain A., Lo J., Harberd N.P., Peng J.R. (2002). Gibberellin Regulates *Arabidopsis* Seed Germination via *RGL2*, a *GAI/RGA-like* Gene whose Expression is up-Regulated Following Imbibition. Gene Dev..

[61] Ravindran P., Verma V., Stamm P., Kumar P.P. (2017). A novel RGL2–DOF6 Complex Contributes to Primary Seed Dormancy in *Arabidopsis thaliana* by Regulating a GATA Transcription Factor. Mol. Plant..

[62] Wild M., Achard P. (2014). The DELLA protein RGL3 Positively Contributes to Jasmonate/Ethylene Defense Responses. Plant Signal. Behav..

[63] Gray J., Caparrós-Ruiz D., Grotewold E. (2012). Grass Phenylpropanoids: Regulate before Using!. Plant Sci..

[64] Lanot A., Hodge D., Lim E.K., Vaistij F.E., Bowles D.J. (2008). Redirection of Flux Through the Phenylpropanoid Pathway by Increased Glucosylation of Soluble Intermediates. Planta.

[65] Zhong R., Lee C., Ye Z.H. (2010). Evolutionary Conservation of the Transcriptional Network Regulating Secondary Cell Wall Biosynthesis. Trends Plant Sci..

[66] Kim W.C., Ko J.H., Kim J.Y., Kim J., Bae H.J., Han K.H. (2013). MYB46 Directly Regulates the Gene Expression of Secondary Wall-Associated Cellulose Synthases in *Arabidopsis*. Plant J..

[67] Bolger A.M., Lohse M., Usadel B. (2014). Trimmomatic: A Flexible Trimmer for Illumina Sequence Data. Bioinformatics.

[68] Kim D., Langmead B., Salzberg S.L. (2015). HISAT: A Fast Spliced Aligner with Low Memory Requirements. Nat. Methods.

[69] Trapnell C., Williams B.A., Pertea G., Mortazavi A., Kwan G., van Baren M.J., Salzberg S.L., Wold B.J., Pachter L. (2010). Transcript Assembly and Quantification by RNA-Seq Reveals Unannotated Transcripts and Isoform Switching During Cell Differentiation. Nat. Biotechnol..

[70] Anders S., Pyl P.T., Huber W. (2015). HTSeq--a Python Framework to Work with High-Throughput Sequencing Data. Bioinformatics.

[71] Kanehisa M., Araki M., Goto S., Hattori M., Hirakawa M., Itoh M., Katayama T., Kawashima S., Okuda S., Tokimatsu T. (2008). KEGG for Linking Genomes to Life and the Environment. Nucleic Acids Res..

